# The prevalence of canine *Leishmania infantum *infection in western China detected by PCR and serological tests

**DOI:** 10.1186/1756-3305-4-69

**Published:** 2011-05-09

**Authors:** Jun-Yun Wang, Yu Ha, Chun-Hua Gao, Yong Wang, Yue-Tao Yang, Hai-Tang Chen

**Affiliations:** 1 National Institute of Parasitic Diseases, Chinese Center for Disease Control and Prevention; the Key Laboratory of Parasite and Vector Biology of the Chinese Ministry of Health; WHO Collaborating Center for Malaria, Schistosomiasis and Filariasis, Shanghai 200025, People's Republic of China; 2 Center for Disease Control and Prevention of Jiuzhaigou County, Jiuzhaigou, Sichuan 623400, People's Republic of China; 3 Department of Biochemistry and Molecular Biology, Wayne State University School of Medicine, Detroit, Michigan 48201, USA

## Abstract

**Background:**

Canine leishmaniasis (CanL) is endemic in western China, resulting in important public health problem. It is essential to evaluate the prevalence of canine *Leishmania infantum *infection for designing control policy. In the present study we report for the first time prevalence of *Leishmania *infection in dogs living in Jiuzhaigou County (Sichuan Provence, China), which is not only an important endemic area of CanL but also a tourism scenic spot, detected by PCR, ELISA and dipstick test. The results could provide key information for designing control programs against canine and human leishmaniasis. In addition, the complete sequence of the *Leishmania *isolate from Sichuan Province has not been reported to date and we present the sequences of 116 base-pair (bp) fragment of the conserved region in the minicircle kinetoplast DNA (kDNA) and the results of phylogenetic analyses based on the sequence of the amplified fragment.

**Results:**

The proportion of dogs infected with *Leishmania *in Jiuzhaigou County was 36.79%, 9.43%, and 51.88% detected by ELISA, dipstick test, and PCR, respectively. The ELISA and PCR tests were more sensitive than dipstick test. The PCR method is the most sensitive way to detect dogs infected with *Leishmania *parasites. The total positive rate for infected dogs in the area was 59.43% by the three methods. The PCR products of 116-bp fragment amplified from the kDNA conserved region of dog blood samples and laboratory maintained *L. infantum *were DNA sequenced and the variation of the sequences was observed. The phylogenetic tree based on the sequences of 116-bp fragment reveals that *L. infantum *is more genetically related to visceralizing species *L. donovani *than to the *Leishmania *species associated with cutaneous disease.

**Conclusions:**

More than half of dogs living in the endemic Jiuzhaigou County were infected by *L. infantum*. Control measures, such as treatment or eradication of infected dogs, or prohibition of maintaining dogs, must be taken against these infected dogs due to their role in the transmission of the infection to vectors. The phylogenetic tree based on the sequences of conserved region in kDNA of *Leishmania *can effectively distinguish species of *Leishmania*.

## Background

Visceral leishmaniasis (VL) is a severe vector-borne parasitic disease of humans and other mammals caused by protozoa of the *Leishmania donovani *complex [1,2]. The disease is endemic in 61 countries and is responsible for the annual loss of an estimated 1.81 million disability adjusted life-years (DALYs) and 57,000 lives [3].Clinically and epidemiologically, there are two main forms: zoonotic visceral leishmaniasis (ZVL) and anthroponotic visceral leishmaniasis (AVL) [4]. ZVL, caused by *L. infantum *or *Leishmania chagasi*, is mainly distributed in Europe, Asia, Africa, and Latin America [5-9]. In most of those regions, canine infection with *L. infantum *is the cause of disease in dogs and as a reservoir for human VL. Accurate and rapid detection of CanL is of great importance to prevent transmission to humans. Clinical diagnosis of CanL is difficult due to its variable symptomatology [10,11] and that it is usually asymptomatic at the early stage of infection [12]. Identification of the infected dogs in endemic areas has major public health implications. It was demonstrated that not only symptomatic dogs but also asymptomatically infected dogs were the sources of the parasite for vector sandflies and as a consequence play an active role in the transmission of *Leishmania *to humans [13-17]. Thus, the evaluation of the prevalence of canine *Leishmania *infection in endemic areas is very important for the epidemiological study and control of leishmaniasis.

VL is still an important public health problem in China and is currently endemic or re-emerging in more than 50 counties in six provinces or autonomous regions in western China including Xinjiang, Gansu, Sichuan, Shaanxi, Shanxi and Inner Mongolia [18-22]. Among them, Gansu, Sichuan, Shaanxi and Shanxi are ZVL endemic areas with dogs as a major reservoir host. In these areas CanL is caused by *L. infantum *infection and transmitted by wild *Phlebotomus chinensis*. The elimination of domestic dogs in endemic areas dramatically reduced the human VL cases, confirming the infected dogs are the major source of the human infection [19]. The data from National Diseases Reporting Information System (NDRIS) operated by the Chinese Center for Disease Control and Prevention (China CDC) [23] revealed that human VL cases increased continuously in these endemic areas during the past years. Lack of information about CanL prevalence in these endemic areas makes it unclear whether the dog infection is blamed to the re-emerge of the disease in the areas of western China. Therefore, determination of canine *Leishmania *infection is important to understand the transmission and to the control of leishmaniasis in the endemic areas.

Serological antibody tesst and PCR detection have been extensively used to investigate canine infection with *L. infantum *[24-28]. Some studies demonstrated that PCR method was more sensitive and specific than parasitological and serological methods [29-31]. Jiuzhagou County of Sichuan Province is not only an important CanL endemic area but also a famous tourism resort. The prevalence of CanL has never been investigated in this area. In this study we investigated the prevalence of *Leishmania *infection in dogs in this county by using PCR detection compared with serological tests. A 116-bp fragment of minicircle kDNA conserved region of different *Leishmania *species was amplified from infected dog blood samples with primers K13A-K13B [32,33] and the PCR products were DNA sequenced. The DNA sequences of this fragment isolated from dogs in this county were compared with the reported sequences of *L. infantum *and other *Leishmania *species isolated from other regions.

## Materials and methods

### Study site

**Figure 1:**
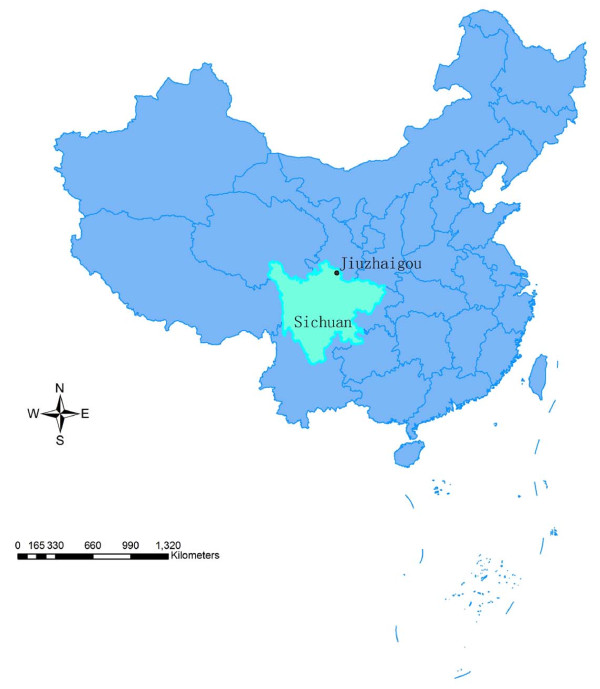
**The map of China showing the location of the study site**. The Jiuzhaigou County is located in the northern part of Sichuan Province in southwestern China.

The study was carried out in the Jiuzhaigou County, Sichuan Province, an endemic area of CanL. The county (32°53' ~ 33°32' N, 103°27' ~ 104°26'E) is located in mountainous area of Sichuan in southwestern China (Figure 1). It has a annual mean temperature of 7.3°C and varied from 16.8°C in the summer to -3.7°C in the winter, with a mean annual rainfall of 700~800 mm. The capital town of the county and several adjacent townships have an altitude of 1,140~2,000 meters above sea level and are visceral leishmaniasis endemic areas with dogs as major hosts. In recent years, about 20 human VL cases were reported per year, and the incidence rate was about 1/2,000.

### Animals and sampling

The protocol for sampling had been reviewed and approved by the Ethical Review Committee of the National Institute of Parasitic Diseases, Chinese Center for Disease Control and Prevention in hanghai. Oral informed consent was obtained from the owners of dogs. About 2,000 dogs lived in VL endemic area of Jiuzhaigou County. For the sample calculation, the formula applied was n = p (1-p) (1.96/D)^2 ^= 97 dogs, with a 10% error level and a confidence level of 95%, where p = 50%. This is estimated based on investigations carried out in other parts of world [25-31]. Based on the calculation 106 household dogs from different breeds were randomly collected in present study at the capital town of the county and adjacent villages in May of 2010. All tested dogs were older than 7-months (going through at least one sandfly season during May to September) and examined for clinical signs of the disease, including dermatological lesions, ocular changes, weight loss, apathy, lymph node, and spleen enlargement. Five ml of blood samples were taken from the foreleg vein of each dog in EDTA-coated polypropylene tubes for isolating parasite DNA for PCR test, and 2 ml of the blood sample was taken for separating sera for the detection of specific antibodies to *Leishmania*. Whole blood and sera were stored at -80°C.

### Dipstick test

The dipstick test, recombinant k39 antigen-based immunochromatographic strip test to detect anti-*Leishmania *antibody (the Kalazar Detect, batch JL1019; InBios, Seattle, WA), was carried out in the field according to the manufacturer's instructions.

### ELISA

An enzyme-linked immunosorbent assay (ELISA) was performed as previously described [24] by using cultured *L. infantum *(MCAN/CN/90/SC) promastigote antigen coating microtiter plates. Sera collected from 23 uninfected dogs with *L. infantum *in the endemic area were tested for cutoff of IgG-specific ELISA determinations (mean plus 3 standard deviations).The sera from two sick dogs with confirmed *L. infantum *infection were used as the positive control.

### DNA extraction

#### (i) Cultured parasite

The *Leishmania *isolate MCAN/CN/90/SC was originally obtained from a dog with leishmaniasis in Sichuan Province and maintained in our laboratory. The parasite was grown in NNN medium at 24°C for 18 days. DNA extraction from cultured parasite was carried out as described by Reale *et al *[26].

#### (ii) Dog blood

The DNA was extracted from dog blood samples using E.Z.N.A. SQ Blood DNA Kit (Omega Bio-tech, Inc.) according to manufacturer's instruction. The extracted DNA was washed with 70% ethanol (vol/vol), and suspended in elution buffer (10 mM Tris, 1 mM EDTA, pH8.0).

### DNA amplification by PCR

*Leishmania *genus-specific oligonucleotide primers K13A (5'-dGTGG GGGAGGGGCGTTCT-3') and K13B (5'-dATTTTACACCAACCCCCAGTT-3') described by Rodgers et al. [30] were used to amplify a 116-bp of fragment in the conserved region of *Leishmania *kDNA minicircles. The primers were synthesized by Shanghai Sangon Biological Engineering Technology & Service Co. Ltd. (Shanghai, China). PCR amplification was carried out as described previously [34] by using Taq DNA polymerase (Promega) and the extracted DNA samples as templates. A positive control containing 10 ng of genomic *L..infantum *(MCAN/CN/90/SC) DNA, and a negative control without template DNA were included. PCR products were analyzed by electrophoresis through 2% agarose gels.

### Cloning and sequencing of 116-bp fragment

The PCR products of 116-bp fragment are too short for sequencing directly and thus cloned into pGEM-T vector using a TA cloning kit (Promega) according to manufacturer's instruction. The recombinant plasmid was sequenced with vector flanking primers T7/SP6 by Shanghai Sangon Biological Engineering Technology & Service Co. Ltd. (Shanghai, China).

### Phylogenetic and molecular evolutionary analyses

DNA sequences were analyzed using GENEDOC (Free Software Foundation, Inc. Boston, USA) and aligned using Clustal X [35]. The phylogenetic relationship of the amplified fragments from endemic dog samples and the 116-bp sequences from different species of *Leishmania *deposited in GenBank was built-up with MEGA version 4.0 [36] and UPGMA [37]. Evolutionary distances were calculated using the method proposed by Jukes and Cantor [38]. Bootstrap analysis [39] was performed with 1000 replicates.

### Statistical analysis

Since true-negative values detected by each test were unknown, in the present study the sensitivity of each test was defined against the total number of samples positive with at least one method following the method described by Lachaud L *et al*. [40].

Epi Info Tm 6.0 (CDC, Atlanta, GA, USA) was used for data statistical analysis by the *X*^2 ^test for the comparison between PCR, ELISA, and dipstick in the detection of CanL.

## Results

### Serological and PCR detection results

A total of 106 dogs, which went through at least one sandfly season, were chosen for serological tests and PCR detection. Six out of 106 (5.66%) dogs presented one or more clinical signs of CanL while other 100 dogs were asymptomatic (94.34%). Results for clinical status, serology (including ELISA and dipstick test), and PCR detection are shown in Table 1. Only five dogs were positive demonstrated by all methods in which three of them showed clinical signs. Thirty-nine of the dogs were positive by ELISA with five of them showed clinical signs. One dog with clinical signs of leishmaniasis was negative by ELISA. Ten of the dogs were positive by dipstick test in which only three of them showed clinical signs. The presence of parasite DNA was detected in 55 dogs, all six dogs showing clinical signs showed positive for parasite DNA.

**Table 1:** Clinical status, serology and PCR detection results in dogs living in Jiuzhaigou County

Group No.	ELISA	Dipstick	PCR		Clinical status	
				
				No. of symptomatic dogs	No. of asymptomatic dogs	Total no. of dogs
1	+	+	+	3	2	5
2	+	-	+	2	25	27
3	+	+	-	0	1	1
4	+	-	-	0	6	6
5	-	+	+	0	3	3
6	-	+	-	0	1	1
7	-	-	+	1	19	20
8	-	-	-	0	43	43

	Total			6	100	106

The sensitivity of each technique was calculated using the total number of samples positive with at lease one method [38]. Thus, the sensitivities of PCR, ELISA, and dipstick test for detection of canine infection with *Leishmania *in the endemic area were 87.30% (55/63), 61.90% (39/63) and 15.87% (10/63), respectively (Table 2). The results showed that PCR detection was the most sensitive method compared to ELISA (p = 0.001) and dipstick (p < 0.001), and ELISA method was more sensitive than dipstick test (p < 0.001).

**Table 2:** Sensitivities of different methods used in the detection of canine infection with *Leishmania *in Jiuzhaigou

	ELISA (%)	Dipstick (%)	PCR (%)	Total (%)
Symptomatic dogs (6)	5 (83.3)	3 (50)	6 (100)	6 (100)
Asymptomatic dogs (100)	34 (34)	7 (7)	49 (49)	57 (57)
Total (106)	39	10	55	63
%	36.79%	9.43%	51.88%	59.43%
Sensitivity*	61.90%	15.87%	87.30%	-

*: Sensitivities were calculated as the number of positive cases of one method against total number of positive cases by at least one method.

### DNA sequence and phylogenetic analyses

**Figure 2:**
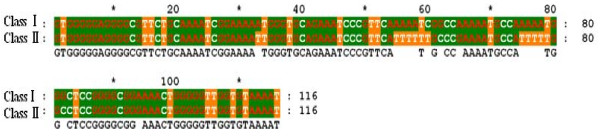
**Alignment of two classes of 116-bp sequences amplified from kDNA minicircles of *L. infantum *MCAN/CN/90/SC**.

Twenty PCR products amplified from dog blood samples and one PCR product from cultured *L. infantum *were subcloned into pGEM-T vector and 10 clones from each PCR product were randomly picked for DNA sequencing. Alignment of the obtained sequences showed that amplified 116-bp sequences contained large variations among the samples from dog blood or from the cultured parasite. Based on the sequence variation two classes of the sequence were divided. The main difference is that two stretches of sequence AAAA in Class I were mutated to TTTT in Class II (Figure 2). Forty more clones from cultured parasite DNA PCR products were sequenced and the variation was confirmed with 80% of Class I and 20% of Class II.

**Figure 3:**
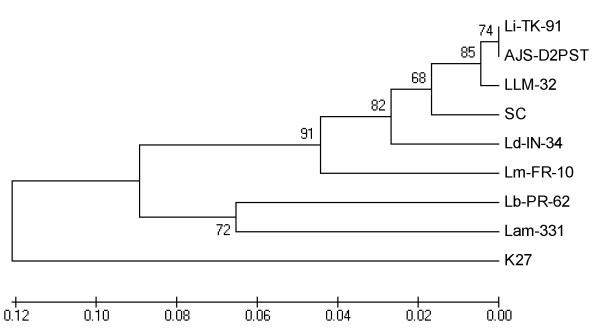
**Dendrogram based on sequence comparison of *Leishmania *spp**. 116-bp fragment in the consverd region of *Leishmania *kDNA minicircles. The dendrogram was constructed using the UPGMA method. Bootstrap analysis was performed with 1000 replicates. *Leishmania spp *used in this tree and their GenBank accession numbers: *L..infantum *MCAN/CN/90/SC (SC, HQ585883); Three *L. infantum *isolates: MCAN/ES/97/LLM-32 (LLM-32, Z35500.1); Li-TK-91 (EU370899.1); AJS-D2PST, (Z35292.1); *L. donovani *(Ld-IN-34, EU370884.1), *Leishmania major *(Lm-FR-10, EU370900.1), *Leishmania braziliensis *(Lb-PR-62, EU370879.1), *Leishmania amazonensis *(Lam-331, EU370875), and *Leishmania tropica *(K27, Z32843.1).

The two classes of 116-bp fragment sequences isolated from the conserved region of *Leishmania *kDNA minicircles from laboratory-maintained *L. infantum *MCAN/CN/90/SC were submitted to GenBank (Bankit) (accession nos.: Class I: HQ585883; Class II: HQ585885). The phylogenetic tree was constructed based on the sequence comparison of class I 116-bp fragment of *Leishmania *kDNA minicircles conserved region among laboratory-maintained *L. infantum *MCAN/CN/90/SC and other *Leishmania *species available in the GenBank (Figure 3). As seen in shown in Figure 2, *L. infantum *isolate MCAN/CN/90/SC used in this study was different from but closely related to the three other existing *L infantum *isolates. However, *L. donovani *and *L. infantum *were linked in a grouping different from the *Leishmanias *associated with cutaneous disease.

## Discussion

Currently, two epidemiological types of VL, anthroponotic (AVL) and zoonotic (ZVL), exist in western China [19,41]. AVL is endemic only in the oases of the plains of Kashi prefecture, Xinjiang Uygur Autonomous Region. Most cases occur in juvenile and adult people. ZVL can be divided into two subtypes, namely mountainous and desert sub-type [22]. The desert sub-type is endemic in the northwestern desert regions of China, including Xinjiang and western Inner Mongolia [22]. These regions were uncultivated deserts for a long time before being populated by immigrants who introduced agricultural activities, consequently, autochthonous infantile kala-azar occurs. This region is considered to be a natural nidus of kala-azar-infected wild animals that are presumably considered to be the source of human infection. The wild species, *Phlebotomus wui *and *Phlebotomus alexandri*, are the vectors infesting different specific landscapes, dry desert region and the stony desert, respectively [22,41]. The mountainous sub-type occurs in the western mountainous and hilly regions of Gansu, Sichuan, Shaanxi, and Shanxi provinces of China. Patients are mostly children younger than ten years old, and infants are commonly infected. These patients are distributed sporadically. The vector of this form is wild *P. chinensis *[22,41]. Elimination and prohibition of dogs in the endemic area markedly reduced the number of human cases, thus dogs are likely the principal source of infection for the mountainous sub-type [18]. Therefore, evaluation of prevalence of *Leishmania *infection in dogs is of great importance to understand the epidemiology of the mountainous sub-type of ZVL and to prevent transmission of human VL. In this study we collected blood samples from dogs in an endemic area of Jiuzhaigou County, China and evaluated the prevalence of *L. infantum *infection in these dogs using PCR, ELISA, and immunochromatographic dipstick tests.

According to our results, among 106 dogs studied, 6 (5.66%) dogs presented CanL signs and 100 (94.34%) dogs were asymptomatic. The seroprevalence of dog infection in Jiuzhaigou County is 36.79% by ELISA, higher than those obtained by various authors throughout the Mediterranean basin [42-44]. However, the positive rate detected by dipstick test (9.43%) is much lower than that detected by ELISA in this study. The highest positive rate (51.88%) was found in the PCR detection of parasitic DNA in the blood samples of dogs with significant higher than that of ELISA (p = 0.001) and dipstick (p < 0.001), indicating the PCR method is the most sensitive to detect *Leishmania *infection in dogs. The result is consistent with that found by other studies [25,45,46].

In epidemiological studies on CanL, serological and parasitic DNA detection are sensitive and specific to detect *Leishmania *infection in dogs [24,25,31,42]. For detection of symptomatic dogs, the two methods obtained comparable sensitivity in this study, while in detection of asymptomatic dogs PCR method was more sensitive than serological methods. The probable reason is that asymptomatic infection dogs may have low infective burden and therefore the lower levels of anti-*Leishmania *antibodies [47] while PCR can detect as few as a single parasite theoretically. In this study, most of dogs (94.34%) were asymptomatic, resulting in lower positive rates detected by serological methods than that by PCR. In addition, asymptomatic but seropositive dogs may indicate previous contact with parasite with some of these seropositive dogs being PCR-negative [42]. The biopsy samples of lymph node, bone marrow, skin or conjunctive were usually taken to detect *Leishmania *infection by PCR and can yield excellent sensitivity [40,42]. Lachaud L. *et al *demonstrated that PCR method targeting kDNA (K13A-K13B) using peripheral blood effectively detected the parasite in symptomatic or asymptomatic infected dogs [48], while other study indicated that blood sample reduces the sensitivity of the test [25]. However, the PCR test based on blood is advantageous than tissues because blood sample can be obtained safely and less invasively, and relatively easy to process. In diagnosis of symptomatic dogs with leishmaniasis rk39 dipstick gave reliable results [49,50], however, our results demonstrated that the test is not suitable to detect asymptomatic infection of dogs with *Leishmania. *The probable reason is that k39 antigen is only expressed during active infections [51,52].

The prevalence of *Leishmania *infection in dogs that we found in Jiuzhaigou County is very high (total 59.43%), especially in asymptomatic dogs up to 57% (57/100). Some studies indicated that asymptomaticlly infected dogs with *Leishmania *were not infective to sandflies [53,54], while some others demonstrated that asymptomatically infected dogs were the sources of the parasite for vector sandflies and as a consequence play an active role in the transmission of *L. infantum *to human [13-17]. The high prevalence of *Leishmania *infection in dogs in regions where leishmaniasis is endemic has to be taken into account in any campaign aiming at controlling CanL. In fact, some authors have demonstrated that removing seropositive dogs is an insufficient method to eradicate CanL [55,56]. In this study we found that *Leishmania *DNA could even be detected in serologically negative dogs. These seronegative dogs are also possible sources of Leishmanial transmission. These data provide a better estimation of transmission level of the parasite in the endemic area, therefore are essential for designing and implementing appropriate control measures.

The results of this study demonstrated that the amplification of 116-bp fragment in the conserved region of *Leishmania *kDNA minicircles could be used for detection of parasite DNA in the blood samples of infected dogs. The sequence data of the PCR products also showed the significant diversity among the samples isolated from different dogs and from lab maintained strain of *L. infantum *(MCAN/CN/90/SC). More than 20% of the amplified 116-bp fragments showed two stretches of sequence AAAA mutated to TTTT and therefore named as Class II compared to the regular Class I type with no mutations. Based on our findings and the findings of others [32,57], it suggests that a certain percentage of sequence divergence existed in the constant region of the minicircle classes not only among species but also within the species or even within the same parasite. It could be due to recombination or mutation events occurring in the kDNA molecules [43]. Based on the divergence existing in the constant region of the minicircle classes among *Leishmania *spp., in this study we constructed a phylogenetic tree revealing that *L. infantum *isolate MCAN/CN/90/SC used in this study was closely related to three other existing *L infantum *isolates, and more genetically related to other visceralizing species such as *L. donovani *than to those *Leishmania *species associated with cutaneous disease. Therefore, the sequence data of *Leishmania *kDNA minicircles could possibly be used for distinguishing species or strains of *Leishmania*. It is the plan to collect parasite samples from different endemic areas of China to genetically type the strains or isolates of *L. infantum *based on the diversities of the *Leishmania *kDNA minicircles

## Conclusions

The results from present study demonstrated that more than half of dogs living in endemic Jiuzhaigou County, China, were infected by *L. infantum*. Control measures, such as treatment or eradication of infected dogs or prohibition of maintaining dogs, must be taken against these infected dogs due to their role in the transmission of the infection to vectors. The phylogenetic tree based on sequences of *Leishmania *kDNA conserved region provides useful way to distinguish *Leishmania *species.

## Competing interests

The authors declare that they have no competing interests.

## Authors' contributions

JYW designed and conducted the study, performed data collection/analysis and drafted the manuscript. YH assisted in field work. CHG, YW and HTC carried out PCR detection and statistical analysis. YTY carried out the immunoassays. All authors read and approved the final manuscript.
